# Attitudes Toward Psychiatry as a Prospective Career among Medical Students in Their Pre-Clinical Year in China- A Pilot Study

**DOI:** 10.1371/journal.pone.0073395

**Published:** 2013-09-02

**Authors:** Xuyi Wang, Xiaojun Xiang, Wei Hao, Tieqiao Liu

**Affiliations:** Mental Health Institute of the Second Xiangya Hospital, Central South University, Changsha, Hunan Province, China; West China Hospital of Sichuan University, China

## Abstract

**Objective:**

To understand the attitudes among medical students in China toward different medical specialties and to find the factors that influenced their choice of career in psychiatry.

**Methods:**

A questionnaire was developed and administered to 287 medical students at the Xiangya Medical College, Central South University in Changsha, China. All the students were asked to rate the importance of five possible factors in choosing a specialty as a vocation: the ability to help patients, interesting and challenging work, lifestyle factors, financial reward, and prestige.

**Results:**

Students reported negative perceptions of psychiatry in regard to all five possible factors that were important in choosing a specialty as a vocation, especially in financial reward and prestige.

**Conclusions:**

Medical students in China have negative attitudes toward psychiatry as a career. Some negative beliefs about psychiatry seem to be due to erroneous or insufficient knowledge that could be corrected during the course of medical education. Some negative attitudes were unlikely to be completely changed until the mental health system in China improves.

## Introduction

Mental health is an essential component of health. Mental disorders can affect not only individual quality of life but also national productivity [1]. Although the burden of mental illnesses has been increasing in all regions of the world, such increase may be more dramatic in China [2], which can partly be understood as a consequence following rapid social changes in recent decades. Unfortunately, mental health services in China are insufficient to respond to the extensive mental disorders prevalent in the country. The shortage of skilled mental health professionals is a major problem: In 2004, the total number of licensed psychiatrists in China was 16,103 (1.24 psychiatrists per100,000 people), which is significantly lower than the global average of 4.15 psychiatrists per100,000 people [3]. The number of mental health institutions and doctors lags far behind the need for mental health services. Also, most general hospitals do not have clinics specializing in mental illnesses, and many clinicians, other than psychiatrists and psychologists, lack awareness and fail to effectively identify symptoms of mental disorders. To cope with this situation, China adopted the nation’s first mental health law in 2012. The aim of the new law was to improve the mental health system in China and protect the rights of patients with mental disorders. For example, the new law requires general hospitals to set up mental health clinics under the guidance of government health departments and requires training of medical workers. The law acknowledged that many more psychiatrists are needed in China.

Previous studies have consistently shown that psychiatry was not the top specialty choice for vocation among medical students in different countries [4–8]. The medical education system in China is different from that in North America and Europe; medical students enroll directly from high school for degrees that require 3 years (for diploma), 5 years (for bachelor), 7 years (for master), or 8 years (for physician); the most common models are 5 and 7 years [9]. Psychiatry is taught in the fourth year of medical school, consisting of 20-30 hours of theoretical lectures and 30 hours of alternating smaller group sessions at clinical sites. At the time of this writing, relatively little study has been done to investigate the attitudes toward different specialties among medical students in China, and to ascertain factors that influence the selection of psychiatry as a vocation.

Although many factors influence a medical student’s career choice of psychiatry and other specialties, attitudes about psychiatrists can play a key role in the declining interest in psychiatry among medical students [7,8,10]. Feifel and colleagues thought there were five possible attitudes that influence the choice a specialty as a vocation, including the ability to help patients, followed by interesting and challenging work, lifestyle factors, financial reward, and prestige [4]. We investigated these five attitudes toward different specialties among medical students in the medical school of Central South University, Changsha, China. The goal of this study was to learn the students’ attitudes and beliefs about different specialties and to find the factors that influenced the career choice by medical students in China, who increasingly eschew psychiatry as a specialty.

## Methods

### Participants

The subjects included in our study were fourth-year medical school students in Xiangya Medical School at Central South University in China. All of them (total number was 344) were invited to participate in the study before the beginning of their rotation in psychiatry. The study was approved by the Central South University Institutional Review Board. All participants were fully informed about the aim of the study. We protected the privacy and confidentiality of all the students. We emphasized that participation was completely voluntary and anonymous. All of them were free to decide whether or not to participate and to withdraw their consent at any time. Participants were assured that their decision would not cause any penalty or disadvantage to their future rotation in psychiatry. All the study participants were asked to complete a questionnaire involving their socio-demographics and their attitudes toward careers in various medical specialties (internal medicine, surgery, obstetrics and gynecology, pediatrics, and psychiatry). The survey took approximately fifteen minutes to complete, and we obtained written consent from all participants.

Two hundred eighty-seven (83.4%, 287/344) medical school students agreed to participate in the survey. Although 287 students participated in the survey, the current analyses include 254 students because some of questionnaires were not completed.

In China, fourth-year students go through most of the required clinical medicine rotations, such as internal medicine, surgery, pediatrics, psychiatry, obstetrics and gynecology, and so on, which gives them some direct experience in different clinical medicine specialties. Such direct experience enables students to gain a limited understanding of specialties.

### Instrument

The questionnaire used to gather information was developed by ourselves and was based on questionnaires developed by others for similar purposes [4]. The survey required 15–20 minutes to complete and consisted of 18 items that explored four areas:

1 The demographic backgrounds of the student, including age, gender, and community size where the students come from.
2 The overall impression of being a clinician as a vocation, which included three questions:
*(1)Which value do you think is important to deciding to be a clinician*? (*You may select multiple choices*):
  a. Being a clinician is a stable career;
  b. Being a clinician indicates a higher social standing
  c. Clinicians can earn more money;
  d. Clinicians are well respected
  e. It is easy to find a position for a clinician.
  f. Other value (Please explain)
*(2) Do you regret going to medical school? Yes/No*
*(3) Do you want to be a clinician after your graduation? Yes/No*
3 The degree to which students were considering possible careers among various medical specialties (internal medicine, pediatrics, surgery, obstetrics/gynecology, and psychiatry); Responses generated for items on both of these sections were assessed on a 4-point scale, with scores ranging among 1 (top choice), 2(more likely),3(maybe) to 4 (unlikely).
  This item is used to reflect the attitudes among medical school students about the various medical specialties.
4 The degree of agreement with a series of statements regarding the importance of five possible factors influencing the choice of a specialty as a vocation.
  (1) Being an expert in the field has enough ability to help patients;
  (2) This is an interesting and challenging career;
  (3) Being an expert in the field will bring a change of lifestyle, so I prefer it;
  (4) Being an expert in the field can earn good money;
  (5) This is a job with good prestige;

Multiple-choice responses to each item were defined as follows: 1(strongly agree), 2(moderately agree), 3(moderately disagree) and 4(strongly disagree).

### Procedure

We created the survey to gain information on medical students’ attitudes toward different medical specialties. Students were given an oral guarantee that their responses to the questionnaire were absolutely anonymous and the participation was completely voluntary. The survey took approximately 15 minutes to complete, and we obtained written consent from all participants. The survey was administered during an orientation session before the start of the psychiatry rotation. The surveyors went to the classroom and distributed the survey forms to every student by hand. All the subjects were required to complete the questionnaire and return it in class.

## Results

The number of valid questionnaires retrieved in our survey was 258. The mean age of respondents was 21 years (SD =1.11). Most of them are Han Chinese. Our findings indicated that most of students reported positive opinions about being a clinician as a vocation. The top three positive opinions about being a clinician were "clinicians are well respected"(86.8%; 224/258), "being a clinician is stable" (83.3%; 215/258), and "being a clinician indicates a higher social standing"(78.3%; 202/258).

Among the 258 students in our sample, most of them chose internal medicine (35.6%) and surgery (31.4%) as their first choice for a career specialty, while few students (only 1.6%) took psychiatry as their top choice. Table 1 summarizes the demographic characteristics of the participants as well as their first career choice (see Table 1).

**Table 1:** Demographic Characteristics of Medical Students (N=258).

	**Percentage (%)**	**Number**
Gender		
Male	61.2	158
Female	38.8	100
Ethnicity		
Han	85.7	221
Other	14.3	37
Career–ﬁrst choice		
Internal medicine,	35.6	92
Surgery	31.4	81
Obstetrics & Gynecology	10.5	27
Pediatrics	8.9	23
Psychiatry	1.6	4
Other	12.0	31

**Figure 1:**
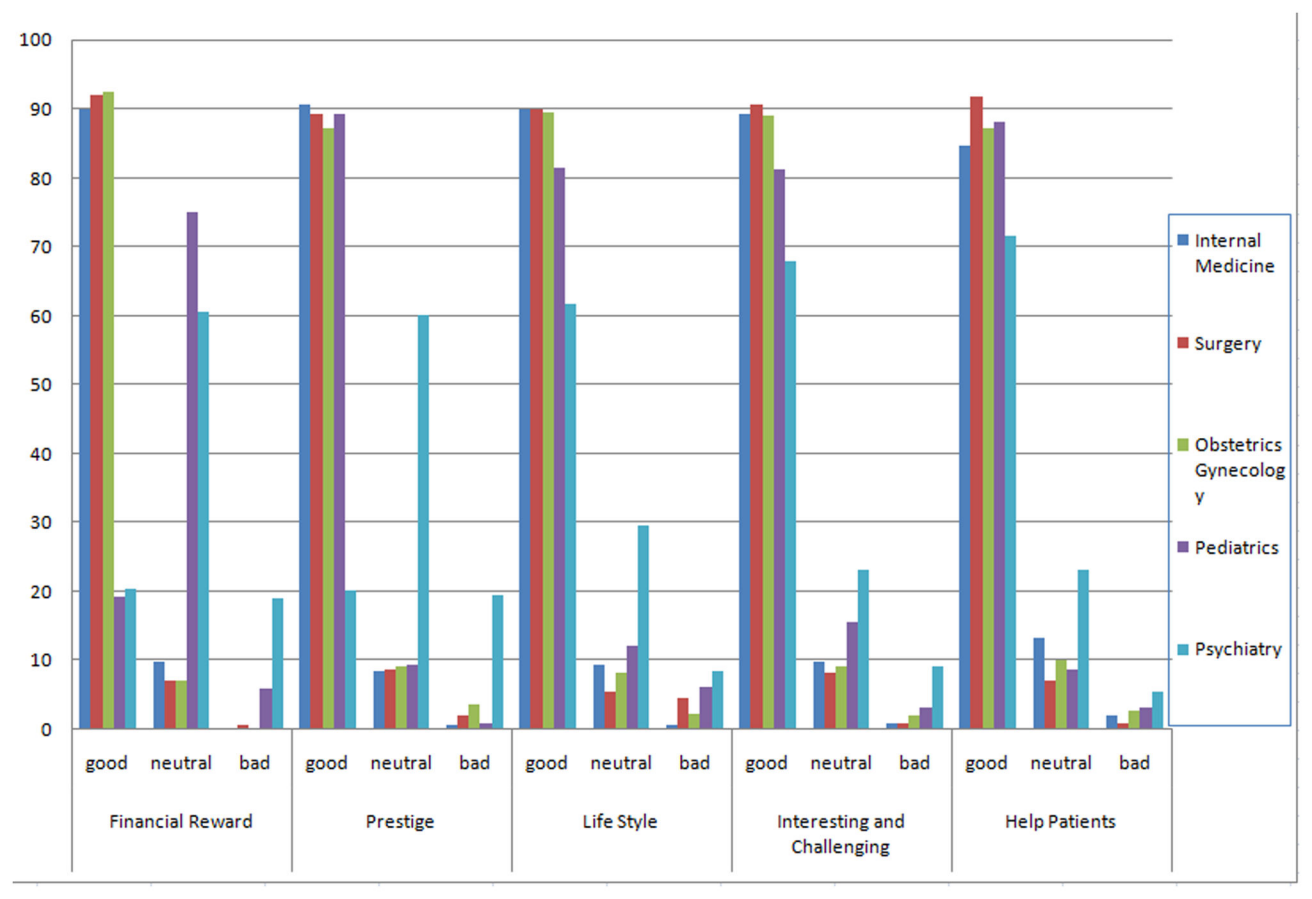
Attitudes toward various specialties as a prospective career among medical school students in China.

Figure 1 illustrates the attitudes of students in terms of the five possible factors regarding various specialties. The students showed negative assessments about psychiatry in all five possible factors that were important in choosing a specialty as a vocation, especially in terms of financial reward and prestige. Only 20.5% of students thought the financial reward of psychiatry was good, while about 90% of students thought financial reward in internal medicine, surgery, and obstetrics and gynecology were good. Only 20.2% of students thought the prestige of psychiatry was good (over 60% of them thought the prestige of psychiatry was neutral), while about 90% of students thought prestige of other specialties was good, including internal medicine, surgery, pediatrics, and obstetrics & gynecology.

## Discussion

### Main Findings

Previous studies investigating the attitudes of medical students toward psychiatry as their future career have consistently shown that psychiatry continues to be an extremely unpopular specialty among medical students [4,10–15]. Our findings indicated that only 4 students (1.6%) among the 258 students in our sample took psychiatry as their first choice of career. This result was consistent with a survey in the USA that revealed that only 0.5% of medical students considered psychiatry as their top choice, whereas 62% of students expressed a negative perception of psychiatry as a career (unlikely or “no way”) [4].

We found negative attitudes about psychiatry among medical students in China in terms of five factor items. With a view to career choice, Chinese medical students were more inclined to see psychiatry as having low social prestige. As far as lifestyle, interesting or challenging job, and ability to help patients were concerned, Chinese medical students thought psychiatrists were not as good as doctors working in other specialties. The consistency of psychiatry’s bottom ranking in all five areas may explain why very few medical students in our sample made psychiatry their first choice of career. Our findings were consistent with previous studies of the attitudes of medical students, which reveal general negative perceptions toward psychiatry [4,11–14,16–18].

### Explanation of Findings

The reasons why very few students made psychiatry their first choice of career were complicated. There is very limited information in China about attitudes toward psychiatry. In China, almost all of the medical schools are funded by the government. After graduation, medical students need to choose specialties and find jobs by themselves [19,20]. All medical students enroll directly from high school; the average medical student is younger than individuals in medical schools in North America. As far as we know, counseling about career choices to the medical school students in China is limited. The career choices of younger medical students tend to be influenced by other people and may reflect the biases toward psychiatry that exist in Chinese society [19,20].

Some negative opinions about psychiatry seem to be due to erroneous or insufficient knowledge, and this inaccurate or incomplete knowledge could be corrected through medical education. For example, students reported that the ability to help patients through psychiatric treatments was poor. In fact, with the advent of newer antidepressants, mood stabilizers, and atypical antipsychotics revolutionizing the standard pharmacological treatment of mental illness, there is sufficiently strong scientific evidence available to suggest that the effectiveness of psychiatric treatments is equal to or superior to that of conventional treatments in other specialty fields [21]. Although there are considerable difficulties in attempting to modify these negative opinions during the process of medical training, evidence indicates that a large proportion of medical students will change their initial career choice at least once within several years [22].

Some of the negative attitudes toward psychiatry seem to reflect the circumstances in China. The rapid shifts over the past three decades in the socio-economic and cultural milieu in China have resulted in changes in the social value system: personal rewards (such as money and prestige) may now play a more important role in how people choose careers [23]. Similar to previous studies from other countries [4,14,18], our findings illustrated that, according to the perception among most medical school students, high prestige and high financial reward are two aspects that a career in psychiatry are not as likely to provide as are other specialties in China. The issues mentioned above provide at least a partial background for why medical students perceive psychiatry as the specialty of lowest status.

### Implications for Practice

Our findings of negative perceptions toward psychiatry among medical school students were obtained only from one medical school in China, so we cannot make more general conclusions regarding the whole country. Thus, our results should be interpreted carefully. Still, results of the present study cause concern. After three decades of rapid economic growth and social development, China’s healthcare system is facing new challenges, such as increased healthcare demands, inadequate healthcare insurance, and inefficient distribution and structures of healthcare resources. The medical resources in China are concentrated in large hospitals and the resources for smaller scale service provision are seriously inadequate [24–27]. The system of healthcare for mental disorders in China has the same problem but is even more pronounced [3,22,28]. Facing these challenges, the Chinese government commenced national healthcare reform in 2009 and adopted the nation’s first mental health law in 2012 [24–27]. To make the national healthcare reform successful, adequate clinical expertise is one of the key factors [3,24,25,29], which meant that more psychiatries were needed in recent years and still are needed in China. More effort should be done to recruit top medical students into the field of psychiatry.

According to the existing literature on the subject, education can produce some changes to medical students’ attitudes toward psychiatry [22,30]. We suggest that all medical schools should consider supplying appropriate education or counseling about career choices to enhance the image of psychiatry (or any other specialty), which would help modify students’ attitudes and opinions toward different specialties and allow a less biased career choice.

Although the community-based mental health service developed very rapidly in recent years, the provision of mental health services is still primarily hospital-based in China. Most general hospitals do not have clinics specializing in mental illnesses, and specialist mental health services remain the predominant component of the system [3,29,31]. Psychiatrists in China are salaried employees. Salaries are made up of base salaries and bonuses. Psychiatrists’ salaries vary greatly according to the work experience, professional level, and hospitals. We suggest that the Chinese government should devote more effort to improve the mental health system by increasing investment, which would encourage people to work in the mental health field. Long-term initiatives are needed to improve public awareness of mental health issues, to reduce the persistent stigma attached to mental illness, and to promote the prestige of psychiatry as a career. We need a better understanding of the issues behind the attitude development of medical students about selection of career specialty.

### Limitations

There are several limitations to our study. First, this was a pilot study, the results were obtained only from one medical school, and our findings might not necessarily apply to the Chinese medical school population as a whole. Future investigation should include more medical schools in China. Another limitation is that only five factors were investigated in our study. More detailed studies, both quantitative and qualitative, should be conducted in order to investigate the attitudes of medical students toward psychiatry.

## Conclusions

Even based on a modest sample size representing a single site, the results of this study provide insights about how medical students in China perceive psychiatry as a career. Some perceptions seem to be due to erroneous or insufficient knowledge and may be corrected during the course of medical education, while some of them are unlikely to change until the mental health system in China improves.
